# Function of caspase-14 in trophoblast differentiation

**DOI:** 10.1186/1477-7827-7-98

**Published:** 2009-09-14

**Authors:** Lloyd J White, Wim Declercq, Frank Arfuso, Adrian K Charles, Arun M Dharmarajan

**Affiliations:** 1 School of Anatomy and Human Biology, Faculty of Life and Physical Sciences, The University of Western Australia, 35 Stirling Hwy Crawley, Perth, Western Australia 6009, Australia; 2 Department of Molecular Biology, Ghent University, Technologie Park 927, B-9052, Ghent, Belgium; 3 King Edward Memorial Hospital for Women (KEMH), 374 Bagot Rd, Subiaco, Western Australia 6008, Australia

## Abstract

**Background:**

Within the human placenta, the cytotrophoblast consists of a proliferative pool of progenitor cells which differentiate to replenish the overlying continuous, multi-nucleated syncytiotrophoblast, which forms the barrier between the maternal and fetal tissues. Disruption to trophoblast differentiation and function may result in impaired fetal development and preeclampsia. Caspase-14 expression is limited to barrier forming tissues. It promotes keratinocyte differentiation by cleaving profilaggrin to stabilise keratin intermediate filaments, and indirectly providing hydration and UV protection. However its role in the trophoblast remains unexplored.

**Methods:**

Using RNA Interference the reaction of control and differentiating trophoblastic BeWo cells to suppressed caspase-14 was examined for genes pertaining to hormonal, cell cycle and cytoskeletal pathways.

**Results:**

Transcription of hCG, KLF4 and cytokeratin-18 were increased following caspase-14 suppression suggesting a role for caspase-14 in inhibiting their pathways. Furthermore, hCG, KLF4 and cytokeratin-18 protein levels were disrupted.

**Conclusion:**

Since expression of these molecules is normally increased with trophoblast differentiation, our results imply that caspase-14 inhibits trophoblast differentiation. This is the first functional study of this unusual member of the caspase family in the trophoblast, where it has a different function than in the epidermis. This knowledge of the molecular underpinnings of trophoblast differentiation may instruct future therapies of trophoblast disease.

## Background

Chorionic villi protrude into the maternal tissue to form a bi-directional barrier with a large surface area for exchange between the maternal and fetal circulations. These villi are covered by cells of the two layers of the trophoblast, the inner cytotrophoblast and overlying syncytiotrophoblast. The cytotrophoblast provides the proliferative pool of cells to fuse with and replenish the overlying syncytiotrophoblast, which is appositional with the maternal circulation. The syncytiotrophoblast is a continuous multinucleated syncytium which structurally and functionally separates the maternal circulation from the fetal tissues. As well as forming a functional barrier, it also fulfils hormonal and endothelial functions and protects the fetus against maternal pathogens [Reviewed in [1]].

Caspase-14 is an unusual caspase expressed in remarkably few tissues, most of which form a barrier between two privileged environments indicating a conserved role in barrier formation [2]. Its characterisation has been best explored in the epidermis where it indirectly modulates corneocyte intermediate filament stability, hydration, water balance and UV-B protection during keratinocyte differentiation through its cleavage of filaggrin [3-6]. However very little attention has been given to caspase-14 in the human trophoblast, although it is present in the placenta [7] and in BeWo cells [8].

Keratinocyte differentiation and barrier formation is dependent on the expression of the Krüppel-Like Factor 4 (KLF4) transcription factor [9-11], which regulates the transcription of cyclin-D2 by binding to its promoter region, leading to cell cycle arrest at the G1/S-phase boundary [12]. It has been noted that KLF4 contains a potential binding motif (YHCD) for caspase-14 [13] and, given the role of both proteins in keratinocyte differentiation and that of KLF4 in the maintenance of the human placenta [14,15], it is attractive to speculate a synergy between the two in trophoblast barrier formation.

The cytoskeletal trophoblast marker cytokeratin-18 is cleaved during trophoblast apoptosis, resulting in the destabilisation of the cell architecture and apoptotic vesicle formation [16,17]. Cytokeratin-18 is a key intermediate filament protein in the trophoblast; the expression, structure and localisation of which remains plastic to modulate the cytoskeletal arrangement in response to a multitude of cell processes. In particular, trophoblast differentiation requires extensive cytoskeletal rearrangement to prepare and exact syncytialisation. That caspase-14 modulates keratin filament stability in the epidermis suggests the potential for a conserved function of caspase-14 in cytoskeletal regulation during barrier formation.

The BeWo cell line was derived from a tumour of the cytotrophoblast known as a choriocarcinoma [18]. Many aspects of trophoblastic growth, differentiation, metabolism and hormone production are observed in this cell line [18]. We have previously shown that BeWo cells treated with 20 micromolar (μM) Forskolin undergo biochemical differentiation similar to that of the human trophoblast, characterised by increased hCG production, after 24 and 48 hours of treatment [8]. We demonstrated that caspase 14 was expressed in these cells. Meanwhile, morphological differentiation, characterised by reduced and diffuse E-cadherin expression symbolising intercellular fusion, is commenced at 48 hours and well established by 72 hours of treatment [8]. Therefore, 24 hours incubation represents early biochemical differentiation, 72 hours represents morphological differentiation, while 48 hours represents the switch between biochemical and morphological differentiation. As such, it is the cell line most representative of in vitro trophoblast differentiation and the model used throughout this paper.

## Methods

All relevant ethical guidelines have been strictly adhered to. BeWo cells were obtained from the American Type Culture Collection (ATCC) and maintained in growth media containing 5% fetal bovine serum (FBS) and 0.5% penicillin/streptomysin in Hams F12K. For treatment, cells were seeded at a density of 2.5 × 10^5 ^cells/ml and grown for 8 hours at 37°C and 5% CO_2_.

### RNA interference (RNAi)

RNAi was conducted by delivering 100 nM of caspase-14 short interfering ribonucleic acid (siRNA) (Invitrogen, Cat. No.1299003) into the cells using Lipofectamine2000 (Invitrogen, Cat. No. 11668) diluted in OptiMEM (Gibco, Cat. No. 31985) as the transfection agent, and incubated for 16 hours. Following this, the growth media was changed for 20 μM Forskolin to induce biochemical and morphological differentiation [19,20], with dimethyl sulfoxide (DMSO) used for normal cycling controls.

### Quantitative real time reverse transcriptase polymerase chain reaction (RT-PCR)

RNA was extracted using the TriReagent method [21]. Briefly, supernatant debris and adherent cells were collected and treated with 500 μl TriReagent (Sigma, Cat. No. T942) and 200 μl chloroform added. Samples were spun, the upper aqueous phase collected and isopropanol added. After overnight precipitation at 4°C, samples were spun, washed with 75% ethanol and resuspended in RNase-free H_2_O. RNA (2.1 μg) was treated with DNase (Promega, Madison, USA) before undergoing reverse transcription (RT) by heating to 25°C for 10 minutes, 55°C for 50 minutes and 70°C for 15 minutes. Following this, cDNA were stored at -20°C until required.

Five microlitres of SybrIQ (BioRad, Hercules, USA) mixed with 1 μl of each primer, 2 μl dH_2_O and 1 μl cDNA was used for all quantitative Real Time RT-PCR (Reverse Transcriptase Polymerase Chain Reaction) reactions conducted. Primers for the homo sapiens mRNA species used are listed in Table 1. Each reaction was denatured at 95°C for 3 minutes and subsequently cycled 40 times at 95°C for 1 second, 60°C for 15 seconds and 72°C for 5 seconds, except for L19, which was annealed at 51°C. Melt analysis was then conducted by raising the temperature from 72°C to 99°C at 0.5°C intervals for 3 minutes to ensure accumulation of the desired product. All PCR data obtained were standardised against the expression of L19.

**Table 1:** Primers for the homo sapiens mRNA species used

**Primer**	**Accession Number**	**Primer Sequence**	**Size**
**Caspase-14**	NM_012114	***Forward ***5'-tgcacgtttattccacggta-3' ***Reverse ***5'-tgctttggatttcagggttc-3'	204 bp
**Filaggrin**	NM_002016	***Forward ***5'-ggcaaatcctgaagaatcca-3' ***Reverse ***5'-tgctttctgtgcttgtgtcc-3'	187 bp
**Keratin-18**	NM_000224	***Forward ***5'-cacagtctgctgaggttgga-3' ***Reverse ***5'-gagctgctccatctgtaggg-3'	164 bp
**KLF4**	NM_004235	***Forward ***5'-cccacacaggtgagaaacct-3' ***Reverse ***5'-atgtgtaaggcgaggtggtc-3'	169 bp
**L-19**	NM_000981	***Forward ***5'-ctgaaggtcaaagggaatgtg-3' ***Reverse ***5'-ggacagagtcttgatgatctc-3'	194 bp
**β-hCG**	NM_001009050	***Forward ***5'-gcaccaaggatggagatgtt-3' ***Reverse ***5'-gcacagatggtggtgttgac-3'	173 bp

### Western blot analysis

Protein was extracted from cells using Radioimmunoprecipitation (RIPA) buffer containing 150 mM NaCl, 50 mM Tris-HCl, pH 7.5, 1% Triton X-100, 0.5% sodium deoxycholate, 0.1% SDS, and 0.1 mM Phenylmethylsulfonyl fluoride (PMSF). The amount of protein was estimated using the Bradford assay [22]. Ten micrograms of protein was denatured by heating to 95°C for 5 minutes prior to separating using 12% sodium dodecyl sulfate polyacrylamide gel electrophoresis (SDS-PAGE), then transferred to a nitrocellulose membrane and blocked with 5% milk powder in 0.05% TBS-T and incubated with the relevant primary antibody in TBS-T overnight at 4°C (Table 2). Membranes were washed in TBS-T and incubated for 90 minutes with a goat anti-mouse IgG (1:10,000) (Sigma, Cat. No. A3688), washed with TBS-T and developed with SuperSignal West Pico Chemiluminescent Substrates (ECL) (Pierce, Cat. No. 34080) as per manufacturer's instructions. Membranes were photographed using a Kodak ImageStation.

**Table 2:** Antibodies used for the detection of proteins via Western blotting

**Target**	**Source**	**Species**	**Size (kDa)**	**Dilution**
Caspase-14	Wim Declercq, Ghent, Belgium	rabbit	29, 20, 18.5	1:2500
β-hCG	Dako; A0231	rabbit	28	1:6000
KLF4	Zymed Labs; 42-4100	rabbit	58	1:5000
Keratin-18	Calbiochem; AP1021	rabbit	44	1:5000
GAPDH	Invitrogen; 39-8600	mouse	42	1:5000

Following imaging, the antibodies were removed from the membranes (DMSO/Forskolin treated; RNAi with DMSO; RNAi with Forskolin) using a Stripping Buffer composed of 1% β-mercaptoethanol in TBS-T for 30 minutes. Membranes were then re-probed with different antibodies to assess protein expression from the same samples. Membranes were reprobed up to 4 times.

Statistical significance for all experiments was determined using One Way Analysis of Variance (ANOVA) t-tests using Microsoft Excel, with statistical significance established as achieving a P-value less than 0.05 (P < 0.05). All experimental groups within this paper contained 6 replicates, and no results were excluded in the preparation of the data. Bar graphs show the Mean +/- the Standard Error of the Mean (s.e.m.).

## Results

### BeWo cell differentiation

**Figure 1:**
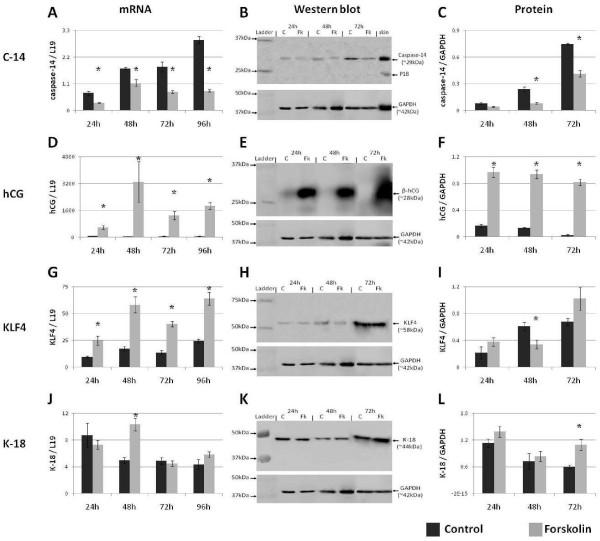
**BeWo cell differentiation effects gene transcription**. The effect of Forskolin treatment on BeWo cell expression of (A-C) caspase-14, (D-F) hCG, (G-I) KLF4 and (J-L) cytokeratin-18. * = P < 0.05 versus controls at the same time point.

Caspase-14 mRNA expression was reduced at all time points following Forskolin treatment (P < 0.05) (Fig. 1A) and caspase-14 protein was also found to be significantly reduced 48 and 72 hours after Forskolin treatment, coinciding with morphological differentiation (P < 0.05) (Figs 1B, C). Our previous studies had shown a fall in caspase 14 mRNA expression with Forskolin, but the timed controls had also shown a concomitant and greater fall, and an increase in protein levels after 24 hours of differentiation [8]. This may be explained by the disparity between the antibodies used, the antibody in the present study being the same as has been extensively used in studies of the epidermis [3,6,23,24]. Interestingly however, cleavage of caspase-14 into its constituent subunits, while observed in the positive control skin sample, was not detected in any BeWo cell extracts regardless of Forskolin treatment (Fig. 1B). This indicates that caspase-14 is not cleaved in the human trophoblast, or is rapidly turned over and present at undetected levels.

Synthesis and production of hCG was significantly induced in BeWo cells by the addition of Forskolin at all time points, confirming the successful initiation of biochemical differentiation (P < 0.05) (Figs 1D-F). Therefore, Forskolin induces BeWo cell differentiation consistent with the human trophoblast.

The cell cycle suppressing transcription factor KLF4 is transcriptionally elevated during BeWo cell differentiation (P < 0.05) (Fig. 1G), suggesting that it is involved in cell cycle withdrawal preceding differentiation. However, Western blot analysis revealed no change in KLF4 protein expression aside from being reduced after 48 hours of Forskolin treatment (P < 0.05) (Figs 1H, I). This implies that while KLF4 transcription is enhanced it is not being translated into protein at nearly the same rate, or that KLF4 protein turnover is elevated. The mechanism responsible for this remains elusive; however, the mRNA and protein data in combination suggest a role for KLF4 in trophoblast differentiation.

The intermediate filament protein cytokeratin-18 (K18) is consistently expressed in BeWo cells. However, after 48 hours of Forskolin treatment corresponding with the onset of morphological differentiation, its transcription was significantly elevated relative to time-matched controls (P < 0.05) (Fig. 1J). K18 protein expression remained stable throughout biochemical differentiation; however, by the onset of morphological differentiation at 72 hours, K18 expression was significantly increased (Figs 1K, L). This suggests that K18 is involved in the cell fusion process. Indeed, the elevated mRNA levels after 48 hours of Forskolin treatment (Fig. 1J) immediately precedes the increase in protein levels (Fig 1K, L). Therefore the increased transcription of K18 follows through into increased translation with morphological differentiation. Consequently, K18 is modulated by morphological trophoblast differentiation.

In the epidermis caspase-14 cleaves and activates profilaggrin. However, while it was present in the epidermal HaCaT cells, no filaggrin mRNA was detectable in either the human placenta or BeWo cell line (See Additional file 1). Therefore, caspase-14 must target discrete cellular pathways in the human trophoblast.

### Validation of RNAi

**Figure 2:**
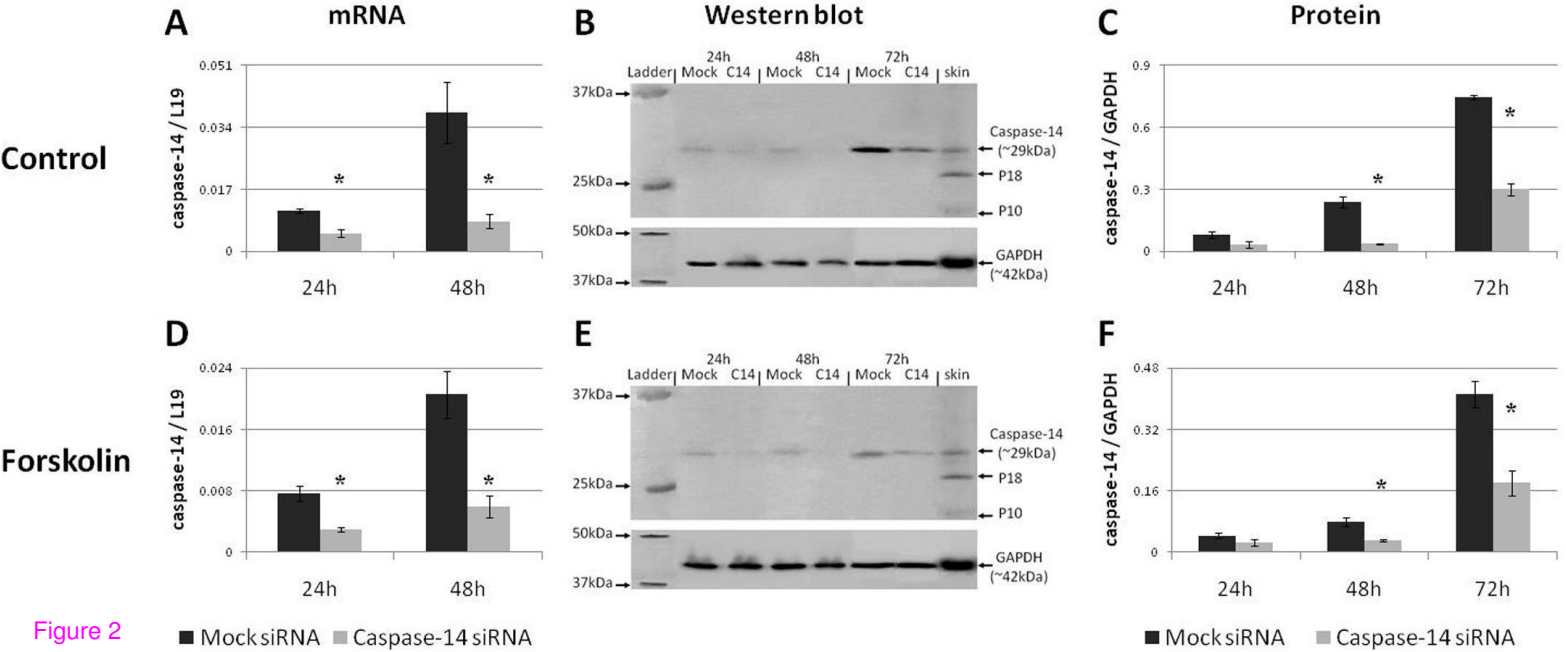
**Suppression of Caspase-14**. Validation of the success of RNAi in suppressing caspase-14 in (A-C) control BeWo cells and (D-F) during differentiation. * = P < 0.05 versus controls at the same time point.

Real Time PCR revealed that caspase-14 mRNA was significantly reduced in DMSO treated BeWo cells at all times compared with those treated with the Mock siRNA (P < 0.05) (Fig. 2A). Furthermore, mRNA expression was reduced at all times following co-incubation with Forskolin (Fig. 2D). Thus, caspase-14 is effectively suppressed in the BeWo cell line following treatment with transcript-specific siRNA.

The successful reduction of caspase-14 protein was quantified using Western blot analysis. Caspase-14 levels were significantly reduced after both 48 and 72 hours of treatment with siRNA in control and differentiating BeWo cells (P < 0.05) (Figs 2B, E). Furthermore, caspase-14 protein in both groups was suppressed to a level approaching significance after 24 hours of treatment (P < 0.1). Therefore, caspase-14 is successfully suppressed in both differentiating and non-differentiating BeWo cells.

As caspase-14 mRNA and protein were similarly suppressed in both control and differentiating BeWo cells (Fig. 2), the addition of Forskolin did not interfere with the efficacy of caspase-14 siRNA. Therefore, caspase-14 is significantly suppressed at both the mRNA and protein levels in both control and differentiating BeWo cells.

### RNAi in control BeWo cells

**Figure 3:**
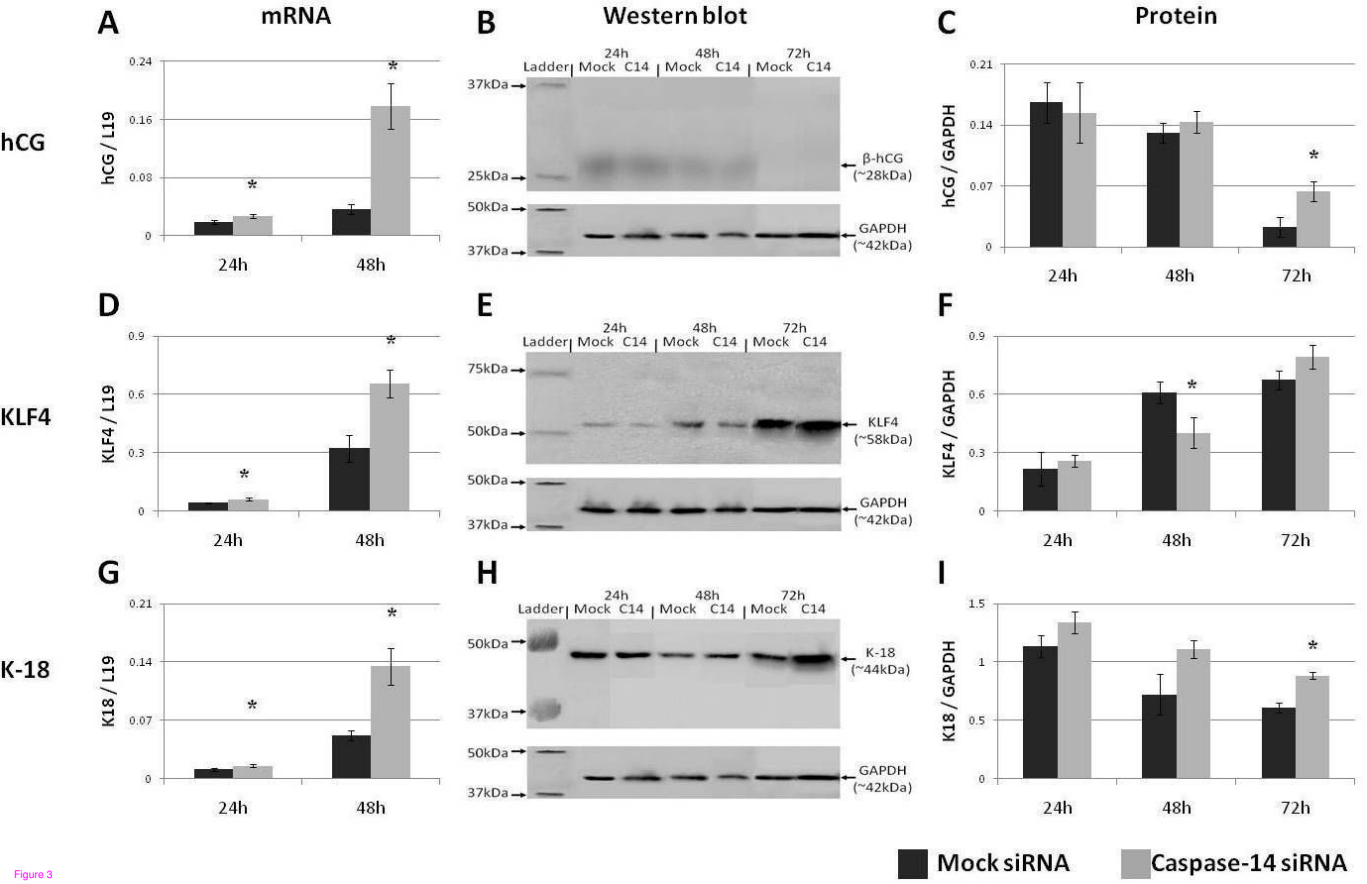
**Caspase-14 modulates trophoblast gene expression**. The effect of caspase-14 siRNA on the expression of (A-C) hCG, (D-F) KLF4 and (G-I) cytokeratin-18 in DMSO-treated BeWo cells. * = P < 0.05 versus Mock siRNA treated controls at the same time point.

Following 24 and 48 hours of culture in the absence of caspase-14, the quantity of β-hCG mRNA was elevated (Fig. 3A). This indicates that transcription of β-hCG is negatively regulated by the activity of caspase-14. Therefore, caspase-14 is biologically involved in the regulation of placental hormone production in control BeWo cells, and may act as a switch for repressing hCG production in the cytotrophoblast. Moreover, as hCG is a key marker of trophoblast differentiation, caspase-14 may be involved in the suppression of this process.

However, it was only after 72 hours of incubation with caspase-14 siRNA that hCG protein was significantly elevated in control BeWo cells (P < 0.05) (Figs 3B, C). Due to the high seeding rate at the commencement of treatment, BeWo cells were confluent after 72 hours in vitro. Since BeWo cells grow as monolayers, confluence may induce contact inhibition of the cell cycle. That β-hCG protein was increased in BeWo cells at this point after caspase-14 knock-down suggests that caspase-14 suppresses hCG production after cell cycle withdrawal in the trophoblast.

KLF4 acts as a transcription factor to suppress the synthesis of cyclin D2, thereby inducing cell cycle arrest at the G1/S phase boundary. Transcription of KLF4 is increased in BeWo cells after both 24 and 48 hours of treatment with caspase-14 specific siRNA (P < 0.05) (Fig. 3D). This suggests that caspase-14 inhibits KLF4-mediated cell cycle suppression in BeWo cells. However, KLF4 translation was not found to be significantly altered after either 24 or 72 hours of treatment but, rather, reduced after 48 hours (P < 0.05) (Figs 3E, F). As with data from differentiating BeWo cells, mRNA and protein data for KLF4 do not correlate with one another. This indicates the presence of post-transcriptional regulation of KLF4 activity in the BeWo cell line.

By suppressing caspase-14, the transcription of K18 was significantly increased after 24 and 48 hours (P < 0.05) (Fig. 3G). This indicates that caspase-14 is involved in inhibiting the synthesis of K18 in the BeWo cell line. No increase was found at the protein level until after 72 hours (P < 0.05), although after 48 hours it was approaching significance (P = 0.06) (Figs 3H, I). This suggests that K18 translation requires a long time period for completion. Alternatively, post-transcriptional mechanisms may inhibit the translation of the mRNA until such time as intermediate filament and cytoskeletal modulation is required.

### RNAi in differentiating BeWo cells

**Figure 4:**
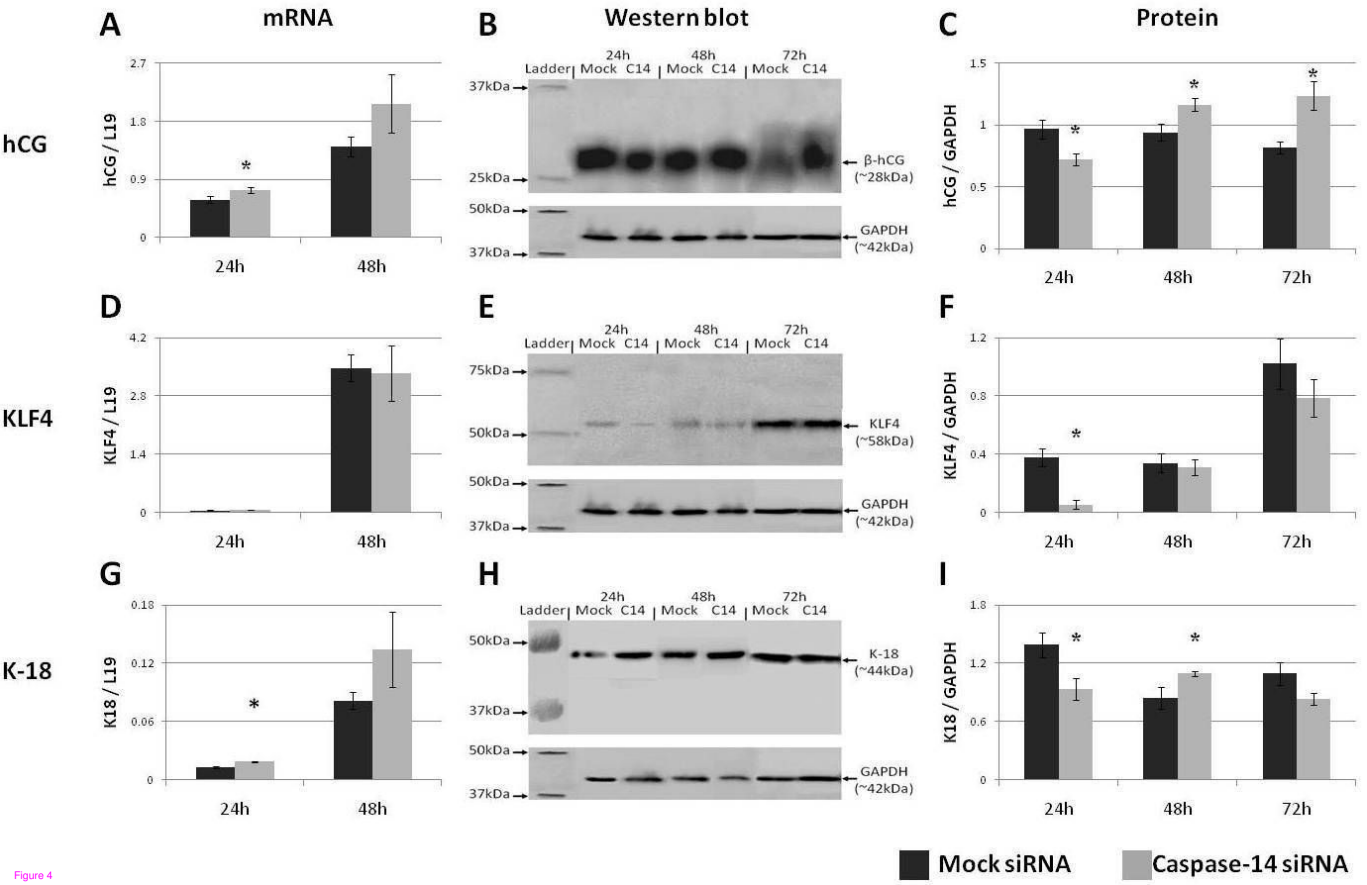
**Caspase-14 modulates trophoblast gene expression during differentiation**. The effect of caspase-14 siRNA on the expression of (A-C) hCG, (D-F) KLF4, and (G-I) cytokeratin-18 in Forskolin-treated BeWo cells. * = P < 0.05 versus Mock siRNA treated controls at the same time point.

In support of data for control BeWo cells (Fig. 3), β-hCG mRNA was increased following the suppression of caspase-14 24 hours after the addition of Forskolin (Fig. 4A), confirming a role for caspase-14 in the regulation of hCG synthesis in the homeostatic and differentiating trophoblast. As hCG production is greatly elevated during differentiation, but with no increase in β-hCG mRNA after 48 hours of siRNA treatment, suggests that there may be a threshold to its production as the level is much higher in these cells than in the non-Forskolin treated cells (>10-fold). This may explain why there is a less marked increase in β-hCG after siRNA in differentiating BeWo cells compared to DMSO treated controls (Figs 3A, 4A).

Nevertheless, β-hCG protein production was increased 48 and 72 hours after Forskolin treatment (P < 0.05) (Figs 4B, C). However, β-hCG protein levels were significantly reduced 24 hours after treatment, representing an interesting regulatory mechanism of hCG production by caspase-14 in the biochemically differentiating trophoblast. Indeed, this may indicate that caspase-14 does not directly affect hCG production but, rather, acts as an upstream modulator of hormone production.

The expression of KLF4 mRNA in differentiating BeWo cells was not significantly affected by the loss of caspase-14 (P >0.05) (Fig. 4D). However, expression of its protein was significantly reduced 24 hours after Forskolin treatment (P < 0.05) (Figs 4E, F). Therefore, caspase-14 only influences KLF4 in differentiated BeWo cells at 24 hours. As KLF4 suppresses proliferation, reduced protein levels indicate that differentiating BeWo cells may be escaping cell cycle withdrawal in the absence of caspase-14, thereby enhancing proliferation. However, this is in conflict with data pertaining to differentiation which suggest that differentiation is enhanced in the absence of caspase-14 (Figs 3A-C; 4A-C). Therefore, a complex mechanism exists in the interaction between caspase-14 and KLF4.

After 24 hours of Forskolin treatment, K18 mRNA levels were increased in response to Forskolin in the absence of caspase-14 (P < 0.05) (Fig. 4G). Furthermore, after 48 hours K18 expression was approaching significance (P = 0.06). Therefore, the lack of caspase-14 during differentiation leads to the increased transcription of this key cytoskeletal component.

Interestingly, the K18 protein was significantly reduced during early BeWo differentiation (P < 0.05) (Figs 4H, I). Moreover, K18 protein was significantly elevated 48 hours after Forskolin treatment in BeWo cells deficient in caspase-14, indicating a period of lag between transcription and translation lasting 24 hours. However, by the time morphological differentiation was occurring (72 hours) expression of K18 was unchanged (P < 0.05), suggesting that caspase-14 does not influence K18 production in fusing BeWo cells. Therefore, caspase-14 modulates K18 production during biochemical differentiation of BeWo cells in preparation for cytoskeletal rearrangements accompanying morphological differentiation.

## Discussion

Induction of BeWo cell differentiation results in reduced caspase-14 expression at both the transcriptional and translational levels (Fig. 1). As the direction of change is the opposite of that observed during keratinocyte differentiation [3,5,23], the mechanism of caspase-14 activation and subsequent function is likely to be different in the trophoblast. Indeed, the cleaved subunits of caspase-14 were not detected in BeWo cell extracts, and the epidermal target of caspase-14 activity, profilaggrin, is absent from the human trophoblast and BeWo cell line (See Additional file 1), confirming a disparity in caspase-14 function between the two tissues.

The reduced expression of caspase-14 throughout BeWo cell differentiation suggests a role suppressing trophoblast differentiation. Analysis of this prospect was conducted by investigating the effect of caspase-14 suppression on hCG, which is commonly used as a marker of trophoblast differentiation and pregnancy. The production of hCG was elevated following caspase-14 suppression (Figs 3A-C; 4A-C), confirming a role for caspase-14 in inhibition of trophoblast differentiation.

The cell cycle inhibitor KLF4 contains a binding motif consistent with that identified for caspase-14 [13]. Furthermore, KLF4 is involved in the maintenance of the human placenta [14,15], while mouse knock-out models of caspase-14 and KLF4 display remarkably similar epidermal phenotypes [6,9,25]. However, the pattern of KLF4 mRNA expression in BeWo cells is the opposite of that for caspase-14, increasing significantly throughout differentiation (Fig. 1G). As KLF4 is a cell cycle suppressor, this indicates that it is involved in the cell cycle withdrawal required for differentiation, suggesting an antagonism between KLF4 and caspase-14 whereby KLF4 promotes while caspase-14 suppresses trophoblast differentiation.

Indeed, suppression of caspase-14 results in increased KLF4 mRNA expression in control BeWo cells (Fig. 3D). As inhibition of caspase-14 results in increased hCG production, indicating enhanced differentiation (Figs 3A-C, 4A-C), this suggests that KLF4 is involved in cell cycle withdrawal resulting in enhanced differentiation. However, despite all of this, there is no concrete evidence for direct interaction between the two, so further examination of the potential relationship between caspase-14 and KLF4 is required.

K18 mRNA and protein were elevated after 48 and 72 hours of Forskolin treatment (Figs 1J-L), correlating with the onset of morphological BeWo cell differentiation. Thus, elevated K18 parallels with the onset of fusion, supporting a significant rearrangement of the intermediate filament cytoskeleton in preparation for intercellular fusion of the trophoblast. That K18 is further increased following caspase-14 suppression (Figs 3G-I, 4G-I) indicates that morphological differentiation is accelerated in the absence of caspase-14. This provides further evidence of a role for caspase-14 in inhibiting trophoblast differentiation. Furthermore, as the cytokeratin architecture is indirectly affected by caspase-14 activity in keratinocytes [6], it is attractive to postulate that K18 arrangement is a downstream target of caspase-14 during the morphological differentiation of the trophoblast.

Interestingly, the transcription of the genes examined in this study appears to be more sensitive to the suppression of caspase-14 than their subsequent proteins (Figs 3, 4). While this may be indicative of the natural delay between transcription and translation, it may also suggest that caspase-14 is involved in suppression of translation within the trophoblast. However, discrepancies between transcription and translation of genes in BeWo cells were also observed in the presence of caspase-14 (Fig. 1), indicating that this is a feature of the trophoblast, not caspase-14.

On the face of it, caspase-14 is a promiscuous molecule. Its suppression seems to affect a multitude of signalling pathways including hormonal (hCG), cell cycle (KLF4) and cytoskeletal (K-18). However, each factor is also implicated in trophoblast differentiation. Synthesis and production of β-hCG and KLF4 is significantly increased throughout BeWo cell differentiation. Moreover, K-18 synthesis and production is increased across the crucial period between 48 and 72 hours when morphological differentiation is induced.

That caspase-14 is significantly reduced across BeWo cell differentiation indicates that it is somehow involved in differentiation, most likely in a suppressive capacity. Indeed, data presented here indicate that caspase-14 does indeed suppress BeWo cell differentiation both in the biochemical (24-48 hours) and morphological (48-72 hours) phases. This suggests an intriguing function for caspase-14 as a checkpoint for adequate BeWo cell differentiation. Future studies into the question of whether caspase-14 regulates the extent of differentiation and acts as an essential checkpoint to prevent uncontrolled differentiation may provide further insight into its function in the human placenta.

In this study there are some differences with our previous results which showed increased caspase-14 mRNA expression during differentiation. However in that study there was a concomitant decline in caspase-14 mRNA expression across the time-course of the study, an effect which was not observed in the present study. Importantly, data obtained from the RNAi experiments are consistent with caspase-14 expression being reduced during differentiation. Furthermore, the differences in caspase-14 protein expression across BeWo cell differentiation between our current and previous studies may be due to the antibody used for its detection. The present study utilised the antibody validated and extensively used to examine caspase-14 function in the epidermis, bringing our data in to line with previous research conducted into caspase-14 biology. Therefore data presented here are more detailed and thought to be more representative of the actual role of caspase-14.

The target of caspase-14 activity in the epidermis- profilaggrin- is not present in either the human placenta or BeWo cell line (See Additional file 1). Therefore, other targets must exist for caspase-14 in the human placenta. Crucially, processing of mature caspase-14 into its active subunits, as evidenced in the epidermis, is absent from BeWo cells. The nature of caspase-14 activity within the trophoblast is therefore uncertain. Despite the significant advances in the understanding of caspase-14 biology that have been made in the current study, its immediate activity and target(s) in the trophoblast remain elusive.

Microarray technology could be used in conjunction with RNAi to reveal genes and pathways directly affected by caspase-14 in the differentiating trophoblast. hCG inhibits the activation of AP-1 transcription factors [26], the latter of which also regulate the transcription of caspase-14 [27]. Indeed the pro-mitotic c-Jun is restricted to the proliferative cytotrophoblast, while the quiescence-maintaining JunD is confined to the syncytium [28]. Thus, c-Jun may stimulate and JunD inhibit caspase-14 transcription in the trophoblast. Further studies involving RNAi against c-Jun and JunD may reveal the mechanisms controlling caspase-14-mediated inhibition of differentiation in the trophoblast. Thereby the molecular triggers dictating caspase-14 activity may be revealed.

## Conclusion

This article reveals a novel function for the protease caspase-14 in modulating the differentiation of the human trophoblast. As inappropriate trophoblast differentiation is a potential cause of the aetiology of preeclampsia and poor fetal growth, further investigation into the formation of the maternal-fetal barrier has important implications for the understanding of diseases in which differentiation may be disrupted. We contend that inappropriate caspase-14 activity during trophoblast differentiation may be involved in the onset of preeclampsia. Further investigation is ongoing to explore this possibility.

## Competing interests

The authors declare that they have no competing interests, and all data are the sole work of the authors. The data herein is unpublished and not under consideration by any other journal. All relevant ethical guidelines have been strictly adhered to.

## Authors' contributions

All original laboratory work and statistical analysis within this research article was completed by LW. Furthermore, the original manuscript was drafted by LW, with vital editing and proofing conducted by WD, FA, AC and AD. AC and AD also equally contributed to the original design and conception of the study. All authors have seen, commented upon, and authorised the submission of the final manuscript.

## Supplementary Material

Additional file 1 Figure five: Filaggrin is not expressed by the human trophoblast. Click here for file
