# Supplementary figures and images for: Function of caspase-14 in trophoblast differentiation
Source: Reprod Biol Endocrinol. 2009 Sep 14;7:98. doi: 10.1186/1477-7827-7-98 (PMC2753366; doi:10.1186/1477-7827-7-98)

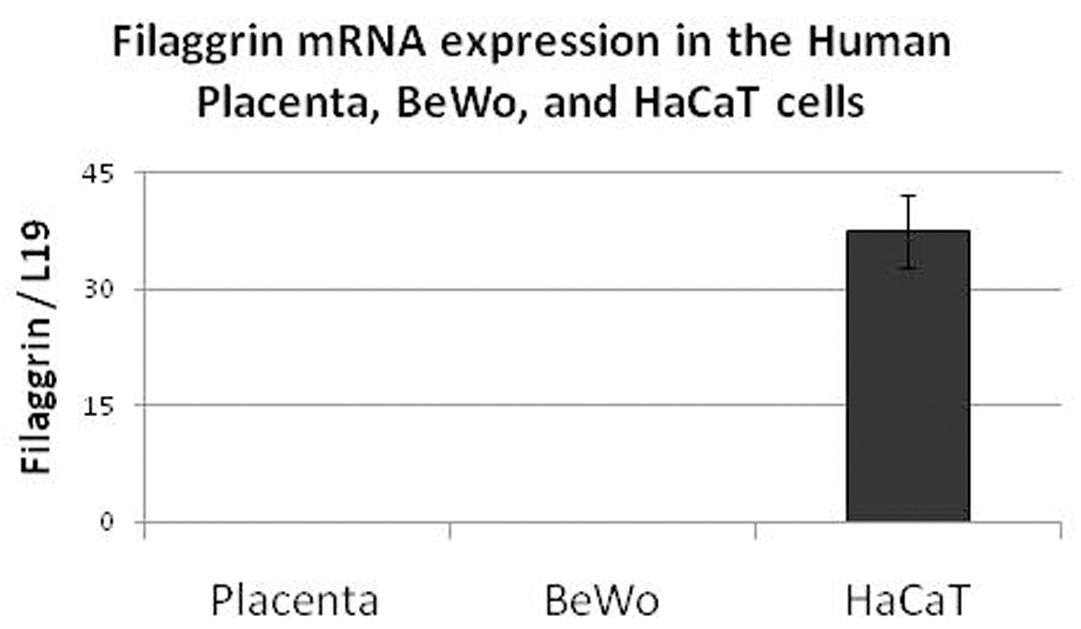

Supplement: Additional file 1 — Figure five: Filaggrin is not expressed by the human trophoblast. [file 1477-7827-7-98-S1.jpeg]
